# A conative educational model for an intervention program in obese youth

**DOI:** 10.1186/1471-2458-12-416

**Published:** 2012-06-07

**Authors:** Jérémy Vanhelst, Laurent Béghin, Paul Stephen Fardy, Gilles Bui-Xuan, Jacques Mikulovic

**Affiliations:** 1 Centre d’Investigation Clinique, CIC-PT-9301-Inserm-CH&U, Lille, France; 2 Unité Inserm U995 & Université Lille Nord de France, Lille, France; 3 Department of Family, Nutrition, and Exercise Sciences (FNES), Queens College, Flushing, NY, USA; 4 ER3S, Université d'Artois, Villeneuve d'Ascq, France; 5 ER3S, ULCO, Dunkerque, France

**Keywords:** Obesity, Physical activity, Intervention strategy

## Abstract

**Background:**

Obesity in children has increased in recent years throughout the world and is associated with adverse health consequences. Early interventions, including appropriate pedagogy strategies, are important for a successful intervention program. The aim of this study was to assess changes in body mass index, the ability to perform sport activities, behavior in the classroom and academic performance following one year of a health-wellness intervention program in obese youth.

**Methods:**

The CEMHaVi program included 37 obese children (19 girls and 18 boys). Participants received an intervention program consisting of physical activity and health education. Assessment included body mass index, academic performance, classroom performance and ability to perform sport activities. Paired t tests were used to assess the effects of intervention, and chi square was used to assess inter-action between measures.

**Results:**

Findings of the study suggest significant decrease in Z scores of Body Mass Index and an improvement of academic performance, classroom behavior and the ability to perform sport activities (p < 0.05). Chi square testing showed significant positive inter-actions between body mass index, classroom behavior and academic performance.

**Conclusions:**

Results following year one of CEMHaVi showed that a program of physical activity and health education had positive effects on obesity, behavior in the classroom and the ability to perform sport activities in obese adolescents. Significant inter-action in changes between variables was observed. Findings are important for designing intervention models to improve health in obese youth.

## Background

Early prevention programs are effective in treating obesity [1-4]. Adolescence, in particular, represents a period during which multiple physiological and psychological changes occur that affect dietary and physical activity habits [5]. Approximately 50% of obese adolescents with body mass index equal to or greater than the 95^th^ percentile become obese adults with serious diseases [6]. Consequences of early obesity include: poor quality of life [7], onset of risk factors that can lead to early pathology [8,9], frequent persistence into adulthood that can lead to increased morbidity of type 2 diabetes, cardiovascular diseases and cancer, and increased early mortality [10,11].

In addition to physical consequences, obesity in youth is also associated with cognitive and psycho-social maladaptive behaviors. Li et al. showed an association between overweight and decreased cognitive function among children and adolescents that leads to poor quality of life, increased illness, incompetence, and low self esteem [12]. Low self-esteem is also associated with a poor academic performance. Obese youth are less able to perform sport activities compared to their lean counterparts further contributing to low self esteem.

Early interventions, including appropriate pedagogy strategies, are important for a successful program. Exercise is one of the factors which contributes to long term weight maintenance [13]. Physical activity, particularly aerobic training, improves lipid oxidation and may be take account in reducing cardiovascular diseases risk factors [14]. Caloric restriction is not recommended in children and adolescents. Children are still growing, diet restriction is not compatible with long term nutrition habits, and dietary restraint for youth as for adults can lead to greater food consumption later [15].

Few studies of diet restriction have been undertaken in children. Protein-sparing and hypo caloric balanced diets have been evaluated for safety and effectiveness for children in outpatient weight reduction [16]. Data suggest that hypo caloric diets are safe and effective in short-term management of pediatric obesity, but should only be undertaken with close medical supervision [16]. Long term effectiveness is uncertain.

Combining diet and physical activity has not been recommended because of the consequences of decreased energy intake with increased energy expenditure. Ebbeling & Rodriguez suggest that exercise offers metabolic benefits for obese children during diet-induced weight loss [17]. Longitudinal studies to assess long term health outcomes are recommended. In our study, we developed a program of physical activity using the Conative Educational Model, i.e. that each subject has an adapted program according to individual competences [18,19]. The principal aim of the Conative Educational Model is to provide a variety of games specifically selected to be enjoyable, maintain interest, and motivate subjects to adhere. The model assures that the child is never in a failure situation which might lead to decreased activity. The child is positioned in an evolutionary dynamics to a particular moment, raising in the same time of his singularity and responsibility, he is an actor of his own construction and management. Each child has an individualized program. This is differentiation pedagogy.

The aim of the present study was to assess changes in ability to perform sport activity, classroom behavior and academic performance following one year of a health-wellness intervention using the Conative Educational Model in obese youth.

## Methods

Thirty-seven subjects, 19 girls and 18 boys from Dunkerque, France participated in the study. Selected physical characteristics are presented in Table 1. Subjects were referred by two pediatricians from the medical community of Dunkerque. Eligibility criteria included: Body Mass Index (BMI) > 97^th^ percentile, 7 to 17 years of age, non-genetic obesity, and a normal clinical examination including normal growth and psychomotor development. Aims and objectives were carefully explained to each subject prior to intervention. Written informed consent was obtained from subjects and parents. The study was approved by the Lille University Research Ethics Committee (Comité de Protection des Personnes, Lille, France). All procedures were performed in accordance with ethical standards of the Helsinki Declaration of 1975, as revised in 2008, and the European Good Clinical Practices. Data were collected at baseline and at the end of year one.

**Table 1:** **Physical characteristics of subjects (*n*** **= 37)**

	**Boys**	**Girls**
**Number**	18	19
**Age (*yr*)**	12.2 ± 2.8	12.7 ± 3.1
**Weight (*Kg*)**	79.5 ± 25.3	78.3 ± 26.8
**Height (*cm*)**	156.9 ± 15.9	155.3 ± 14.6
**BMI (*Kg/m²*)**	31.3 ± 6.1	31.1 ± 6.1

### CEMHaVi intervention

CEMHaVi is a unique program of exercise and health education developed by Applied Physiology Laboratory of University of Littoral Cote d’Opale (France, Nord) and Dunkerque medical community.CEMHaVi consisted of one hour every three months for health education and two hours per week of physical activity in the university gymnasium using the Conative Educational Model [18,19].

### Health education

Health education was adopted from PATH (Physical Activity Teenage Health) translated into French [1,20]. A manual consisting of written and oral exercises on the following topics was provided to all subjects: 1. Function of the heart, cardiovascular anatomy and physiology, cardiovascular risk factors, and the effects of obesity, 2. Physical activity, prescribing exercise, exercise training and the benefits of physical activity, 3. Proper nutrition, macro-and micro nutrients, the importance of water, saturated fat and cholesterol, food guide pyramids, and the importance of eating breakfast, 4. How to deal with stress, explaining stress and stressors, identifying positive and negative stress, stress management techniques, and the effects of relaxation and physical exercise on stress, and, 5. Sleep and the consequences of sleep deprivation with obesity.

### Conative educational approach

The conative educational approach is based on the idea of conative curriculum which models a sense given by to one’s actions [19]. Identifies five *impetus* which lead one’s actions. This model allows the resarcher to identify conative stages through observation of child’s practice, and to assess progress or evolution in the three components (structural, functional and technical) forming the model. The summation of these three components is called “guiding principle” which organize five conative stages, from novice (1) to expert (5) [19]. Subjects identify their stage and observe their progress in the practice of sport. Progress of the intervention program is associated with a teaching activity adapted to the stage that best encourages acquiring of new skills and progressing from one stage to the next. Subjects were evaluated individually and the activity program was adapted for each subject at the stage that met the subject’s ability.

### Measurements

#### Anthropometric measures

Body mass was measured without shoes and heavy outer garments to the nearest 0.1 kg using an electronic scale (Oregon Scientific®, GA 101, USA). Height was measured without shoes to the nearest 0.1 cm using a standard physician’s scale. Body Mass Index (BMI) was calculated by Weight (kg)/Height (m²), and was converted to Z-score of BMI.

#### Ability to perform sport activities (conative curriculum)

Ability to perform sport activities was assessed by only a CEMHaVi researcher with scales for team sports, e.g. soccer, handball, basketball, etc. and net sports, e.g. tennis, badminton, volleyball. The five levels in conative curriculum are described in Table 2.

**Table 2:** Description of conative curriculum criteria for each sport topic

	*Team sports*	*Net sports*
**Stage 1**	It is the cluster phenomenon. There is no distinction between attack and defense. Children flock to the ball without knowing what to do.	It is the returner. The child will return the ball to the other side of the net regardless of where it will land. He sees the ball as an obstacle.
**Stage 2**	There is a structure of defense, but it is primary ("You are left in defense, you do not move and defend yourself!"). The child begins to understand the system Attack/Defense. It fits the more often a zone defense.	It is the underwriter. The child will place the ball on the other side of the net, but it does not replace are after firing. It will search the free space.
**Stage 3**	Personal gaming, sports spectacle. The child will attempt to technical moves to draw attention, to do the show. He tends to play alone, and most often he loses the ball.	Technical application (smash). The child will place the ball, then it will be replace after firing.
**Stage 4**	Technical-tactical sequence. The child makes tactical decisions. They develop a strategy. This stage is very difficult to acquire.	Technical-tactical sequence. The child will develop tactical plans, strategies of attack and defense. Stage difficult to acquire.
**Stage 5**	This stage is represented by the expertise in the practice.	This stage is represented by the expertise in the practice.

#### Academic performance and classroom behavior traits

Academic performance was assessed by questionnaire interview between a researcher from CEMHaVi and the teacher. The questionnaire was comprised of two components: 1) notation and, 2) life in the classroom. The notation score was calculated from the formula: general mean of pupil/general mean of classroom × 100. The individual pupil mean of note includes different disciplines, e.g. mathematics, physiology, French, etc. Each pupil has a note score in each discipline between 0 and 20. In the end, the teacher sums the note of each discipline and calculated the general mean of pupil. According to the general mean of class, the pupil has a score class ranking. Classroom performance consisted of measures of self-esteem, self evaluation of academic performance, and ability to work alone. Interviews were scheduled with the teacher at baseline and following year one for each subject.

### Statistical analysis

Data were analyzed using the Statistical Package for the Social Sciences, Windows 11.5 (SPSS Inc., Chicago, IL, USA), Excel 2003 (Microsoft Inc., Redmond, WA, USA) and Sphinx (Chavanod, France). Means were calculated at baseline and following intervention and were compared by paired *t* tests to assess the effects of the intervention program. Measures included academic performance, Z-score of BMI, and the stage of conative curriculum. The chi square test was used to assess the interactions between these three variables during the intervention program. A *p* values < 0.05 were taken to be significant with a 95% confidence interval.

## Results

Significant improvements were observed in Z score of BMI and ability to perform team and net sports (Table 3), and behavior traits in the classroom, i.e. self-esteem, self evaluation of academic performance, and ability to work alone (Table 4). A non-significant increase was observed in academic performance based on class rank.

**Table 3:** Pre and Post intervention mean differences in Z score of BMI and the stage obtained in conative curriculum (n = 37)

	**Pre intervention**	**Post intervention**	**Δ**	**Δ (%)**
Z score of BMI	5 ± 1.9	4.5 ± 1.9*	- 0.4	- 8.7
Conative Curriculum				
*Team sport*	1.3 ± 0.5	3 ± 0.5*	+ 1.7	+ 90
*Net sport*	1.3 ± 0.6	3 ± 0.7*	+ 1.7	+ 52.9

* p <0.05.

**Table 4:** Pre and Post intervention mean differences in academic performance and the classroom behavior (n = 37)

	**Pre intervention**	**Post intervention**	**Δ**	**Δ (%)**
Changes in class standing (*%*)	96.7 ± 18.7	98.1 ± 22.9	+ 1.4	+ 1.5
Self esteem (*/12*)	6.2 ± 2.7	9 ± 2.7*	+ 2.8	+ 35.1
Self evaluation of academic performance (*/16*)	9.1 ± 3.7	12.4 ± 2.7*	+ 3.2	+ 45.5
Ability to work alone (*/16*)	9.4 ± 3.8	12.4 ± 3.3*	+ 3	+ 31.9

* p <0.05.

Significant chi square findings suggest an interaction between decreased Z-score of BMI and improvement in classroom behavior traits (^2^ = 57.14, df = 2, *p* < 0.001), increased ability to perform sports and improvement of academic performance, (^2^ = 62.65, df = 2, *p* < 0.001), and increased academic performance and improved classroom behavior traits (^2^ = 10.34, df = 2, *p* < 0.001). Finally, a strong interaction was found between decreased Z-score of BMI and increased in sport activity (^2^ = 18.40, df = 2, *p* < 0.001).

## Discussion

To the best of our knowledge, the present study is the first to assess changes in academic performance, classroom behavior and ability to perform sport activities following a one year health-wellness program in obese youth using the Conative Educational Model. Results of the present study suggest that the conative model had a positive impact and that a strong inter-action exists in changes among measures.

Significant decrease in Z score of BMI was important because of the association between obesity and cardiovascular disease, metabolic syndrome or type 2 diabetes [21]. Several studies of intervention programs treating obesity in youth have been undertaken [22-26]. Although the studies have dissimilar methodologies most showed decrease in BMI. A limitation of these interventions was the short duration of programs. CEMHaVi was planned for 12 months to allow for a greater impact on healthy lifestyle than reported previously.

Krukowski et al. suggest that psychosocial variables, such as weight-based teasing, be considered in future research examining the impact of childhood obesity on school performance and the effect of health intervention studies [27]. Increased body weight is independently associated with decreased visuospatial organization and general mental ability among children [12]. Obesity in adolescence is also linked with poor physical quality of life [7]. Another goal of CEMHaVi was to assess psycho-social issues because of their relationship to feeling ill, incompetence, and a low self esteem. Hillman et al. suggested that fitness may be related to better cognitive functioning in preadolescents and may have implications for increasing cognitive health in children and adults [28]. The present study confirms the findings of Hillman demonstrating a strong inter-action between a decreased Z score of BMI and improved of classroom behavior, and increased academic performance (changes in class standing). Decreased BMI also had a favorable impact on self-esteem and the self-assurance. The present study demonstrates the inter-actions of physical and psycho-social measures and the contribution of physical activity to their improvement.

To reinforce self esteem and classroom performance, the CEMHaVi program used the Conative Educational Model [18]. Exercises during physical activity sessions were programmed as a function of subjects individual abilities, i.e. stage obtained in the conative curriculum. In the conative model, obese youth are never in a situation of failure prior to the activity, but rather value activity in relation into their effort and ability. Findings of CEMHaVi showed that the decreased BMI is associated with improved of ability to perform sport activity. Increased ability to perform, in turns, is associated with improved academic performance. Obese youth feel better during exercise sessions. As a consequence subjects are motivated to do more physical activity and to increase the cognitive function [28].

One of the limitations of the present study is the absence of a control group. Because of time limitations and financial constraints a randomized control group was not possible. However, the study has pre-test/post-test design and the group participants act as their own control subjects. Nevertheless, it would be preferable to develop this program with a control group in order to have more rigorous methodology, and to increase the number of participants for statistical power. Therefore, the present study is a pilot and its findings are preliminary, but encouraging.

## Conclusions

Results following year one of CEMHaVi showed a close relationship between BMI, academic performance and ability to perform sport activity. Of greatest importance is that Z-score of BMI decreases proportionally to improved behavior and quality of life in classroom and ability to work alone. Results also showed a positive inter-action between academic performance and the ability to perform sport activities. Finally, the positive effect of academic performance on behavior traits in the classroom demonstrates in obese youth that increased academic performance results in better integration into classroom. These results are particularly favorable for obese youth to improve their general health state, both physical and mental.

## Competing Interests

The authors declare that they have no competing interests.

## Author contributions

J. Mikulovic, L Béghin, J. Vanhelst conceived the idea. J. Vanhelst and P Fardy wrote the first and subsequent drafts. G. Bui-Xuan helped developed the ideas. All authors read and approved the final manuscript.

## Funding

There are no funding sources for the work.

## Pre-publication history

The pre-publication history for this paper can be accessed here:

http://www.biomedcentral.com/1471-2458/12/416/prepub
